# A study of grief experience interviews with family members of patients with advanced malignant tumors based on noninvasive death index monitoring

**DOI:** 10.1186/s40359-023-01396-9

**Published:** 2023-10-26

**Authors:** Li Ren, Liang-liang Shen, Hong Lu, Xiao Wen, Chun-juan Xu

**Affiliations:** 1 Medical Oncology, Peking University International Hospital, Life Park Road No.1 Life Science Park of Zhong Guancun, Chang Ping District, Beijing, 102206 China; 2 Beijing Health Vacational College, Beijing, 101100 China

**Keywords:** Non-invasive mortality index, Family members of patients with advanced malignant tumors, Grief experience interview

## Abstract

**Objective:**

To explore the grief experiences of family members of patients with advanced malignant tumors before and after death.

**Methods:**

This study used both quantitative and qualitative research methods. A total of 10 people with family members with terminal malignant tumors were chosen and assessed five times according to a specific non-invasive mortality index and the Distress Thermometer scale. Additionally, the participants attended an in-depth interview.

**Results:**

The grief experiences of the bereaved included their knowledge of and attitude towards death, the physical and mental conditions of the family members of patients in the terminal stage, the needs of family members, and the response to death and growth of those family members.

**Conclusions:**

The grief experience interviews of family members of patients with advanced malignant tumors are universal. It is suggested that the nursing staff should pay attention to the emotional experience of the bereaved after the death of the patient throughout the whole nursing process, including the continuous follow-up during the home period. It is hoped that the implementation of grief counseling methods in the later stage can help the bereaved to successfully go through the grieving period, prevent grief disorders, and help them return to society.

## Introduction

The total population of China (as a populous country) accounts for about one-fifth of the world’s population; the number of new cases of malignant tumors accounts for 22% of total global cases, and the number of deaths accounts for 27% of total cases [1]. Often, the death of a family member brings great distress to people, causing physical and psychological issues and impacting social relations [2]. The death of patients with tumors will cause severe grief for family members, significantly lowering their immune status and leading to a series of physical and mental reactions. In China, oncology nursing focuses on patients with tumors and neglects the psychological feelings of family members. Despite the many research directions on grief counseling, no systematic guideline exists [3–6]. Domestic and international studies have revealed that following the death of a patient, the family of the deceased experience sad reactions and health problems in the near and long term. It is believed that effective support can help individuals maintain emotional connections, access help, spend some time, and distract themselves [7].

In view of China’s national conditions, people are afraid to face death and talk about death, and family members have no way to express their inner sadness and emotional needs. Therefore, based on the time point of occurrence of specific noninvasive death indicators, this study conducted grief experience interviews, conducted Distress Thermometer (DT) measurement to explore the real grief experience of family members, and real-time tracked the grief status of family members in the later period after the death of patients. In order to lay the foundation for the next implementation of grief counseling, the real implementation of the whole process of human care, reflecting humanistic care.

## Subjects and methods

### Selection of subjects

The objective sampling method was used to select the family members of patients with advanced malignant tumors who were hospitalized in the medical oncology ward of our tertiary hospital in Beijing from June 2018 to August 2019 as the subjects.

**Fig. 1:**
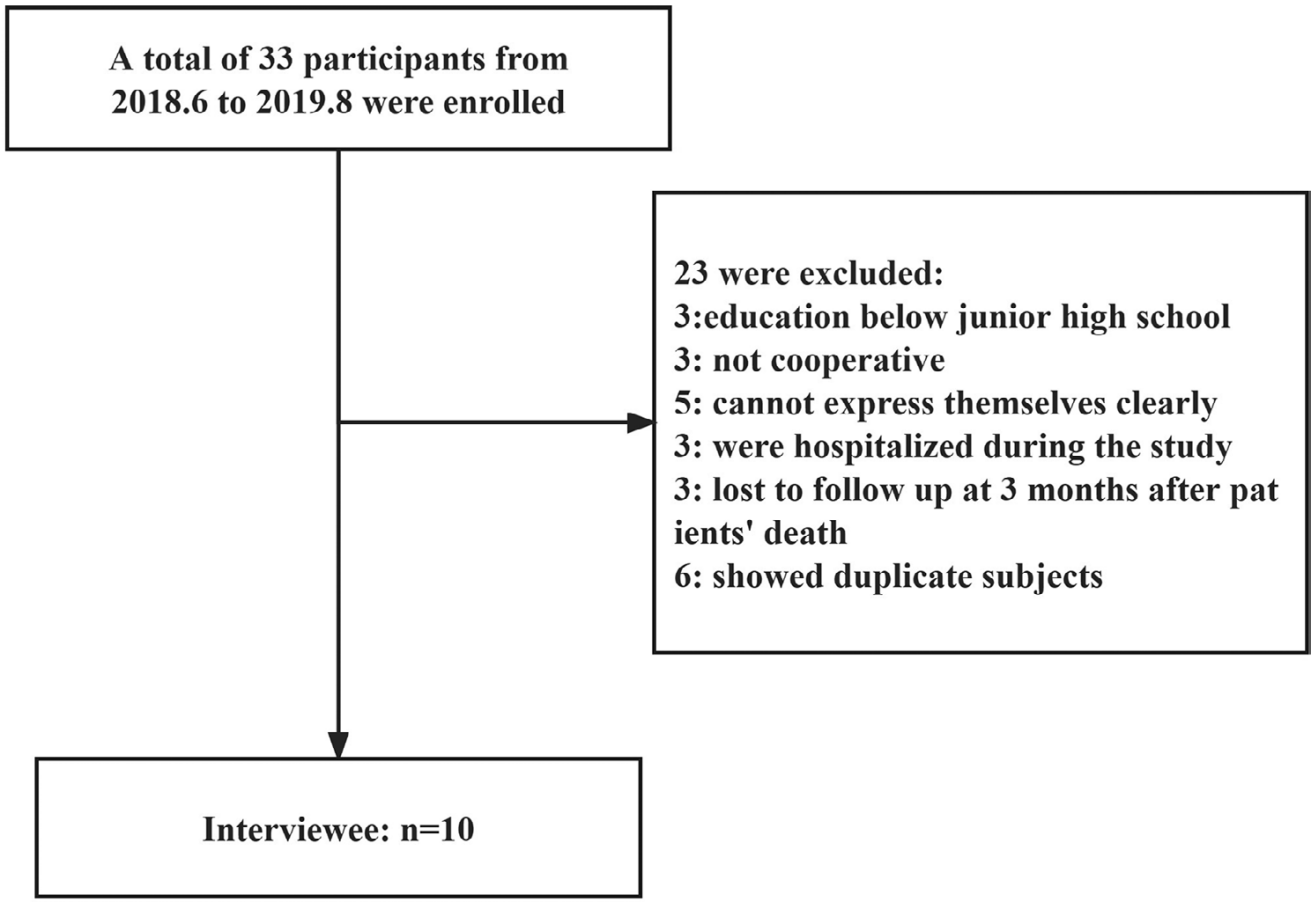
Details about the sampling procedure

The inclusion criteria were: (1) Family members of terminally ill patients living in the city confirmed by pathology or cytology (2) Family members with a junior high school education or above (3) family members with communication, cognitive disability or no mental illness (4) family members who take care of the patient for the longest time every day (5) voluntary participation after informed consent; Exclusion criteria: (1) The family members’ education level is below junior high school (2) the family members are not the author (3) the family members are unable to clearly express their inner feelings (4) the family members who were sick and hospitalized during the investigation (Fig. 1).

The subjects were identified using the letters “A” to “J.” They were aged between 31 and 72 years, with an average of 48.7 years, and included 6 males and 4 females. Among them, 4 were educated to junior college level, 3 had a bachelor’s degree, 2 had a master’s degree, and 1 was educated to technical secondary school level, respectively.

### Methods

#### Specific indicators of death

The case information of 77 patients who died in the medical oncology department of our hospital from December 23, 2015, to January 30, 2018, was analyzed retrospectively via a systematic literature review. Thirteen clinical symptoms and signs that could be used to predict the death of patients within 3 days were identified, including the disappearance of the pupillary reflex, weakened response to verbal stimulation, weakened response to visual stimulation, inability to close the eyelids, downwards movement of the nasolabial fold lines, neck hyperextension, loud snoring, upper gastrointestinal hemorrhage, peripheral cyanosis, mandibular movement during breathing, 24-h urine volume < 100 mL, chilling of extremities, and abnormal respiratory rhythm and frequency. Among them, the symptoms and signs of 66 patients concentrated on 8 aspects of the 13 indices. Based on the analysis results, 8 indices were selected (from the 13 indices) as the ultimate specificity reference indices to forecast the death of patients within 3 days. These specificity indices provide a clinical basis for predicting the time of death.

#### Quantitative research

According to the time node of clinical death indices, the Distress Thermometer (DT) scale, which was developed by Roth in 1998 [8], was used to evaluate 10 subjects a total of five times: at hospitalization, 3 days before death, 1 month after death, 3 months after death, and 6 months after death. Table 1 presents the general information and evaluation results of the patients and their family members.

**Table 1:** General data and evaluation results of patients’ family members

No.	Gender	Age(years)	Occupation (Industry)	Relationship with the patient	Education background	DT First measurement (after hospital admission)	DT Second measurement (3 days before death)	DT Third measurement (1 month after death)	DT Fourth measurement (4 months after death)	DT Fifth measurement (6 months after death)
A	Male	≥ 60	Transportation industry	Father-son	College or University	3	8	7	5	3
B	Female	45–60	Medical insurance industry	Couple	Secondary school or less	2	7	6	4	1
C	Male	≤ 45	Service industry	Couple	College or University	3	10	8	5	3
D	Female	45–60	Service industry	Couple	College or University	7	7	8	6	4
E	Female	45–60	Culture/Sport/Education industry	Couple	Master’s degree and high	1	6	5	3	1
F	Male	45–60	Culture/Sport/Education industry	Couple	Master’s degree and high	4	8	6	4	2
G	Male	≤ 45	Information technology industry	Couple	College or University	9	8	6	5	2
H	Male	≥ 60	Banking/Finance industry	Couple	College or University	7	9	10	7	5
I	Male	≤ 45	Others (e.g., Government or non-profit organizations)	Mother-son	College or University	2	10	10	10	9
J	Female	≤ 45	Others (e.g., Government or non-profit organizations)	Father-daughter	College or University	2	7	10	7	4

#### Qualitative research

##### Sampling method

Interview is one of the important data collection methods for qualitative research, whose purpose is to understand the real inner experience of family members when facing the death of patients. Objective sampling method was used to select qualified respondents according to inclusion and exclusion criteria for individualized in-depth interviews. Pay attention to the selection of family members who are highly expressive and can represent the characteristics of the study group.

The sample size of this study was determined according to the repeated appearance of data provided by the respondents and no new theme during the data analysis (data saturation) [9].

##### Interview questions

Before preparing the outline of the interview, questions were prepared preliminarily according to the relevant literature, and the questions were discussed, analyzed, and modified by a grief counseling group. In the preliminary experiment, pre-interviews were held for the family members of one patient with an advanced tumor, and the interview’s outline was modified again in accordance with the analysis results of the interview. The outline covered the following six open-ended questions: ① How do you think about life and death? ② How is your relationship with the patient? ③ How would you feel if your family member died? Could you talk about your feelings? How would the death of your family member affect your life and psychology? ④ Which do you think affects you more: your family member’s current situation or their death? ⑤ What do you think you can do to ease the pain of bereavement? ⑥ Have you ever experienced a bereavement? How did you deal with it?

##### Ethical principles

This study was approved by the ethics committee of our hospital, and it did not impact the physiology and psychology of the respondents during the research process. After obtaining informed consent from the respondents, the investigators began recording the formal interviews. At the end of each interview, the investigators expressed their gratitude to the respondents, embraced them, and gave them small gifts.

##### Data analysis

Data analysis is carried out according to the 7-step analysis method of Colaizzi [10], which includes: transcribe, analyze, encode, classify, extract themes, find associations and check the authenticity of data. Data analysis was performed by two researchers. On the basis of continuous and repeated analysis of the same data independently, the two people constantly compare with the original data. Try to avoid adding the researcher’s own theory and experience in the analysis, follow the information provided by the original materials, and constantly reflect, experience and find the theme in the data.

## Results

The semi-structured interview results for the family members of 10 patients in the terminal stage are given below. Among the 10 participants, 5 were female, and 5 were male. Seven were spouses of patients; 4 were husbands and 3 were wives. One, who was the father of a patient, had a father–son relationship. Another, who was the son of a patient, had a mother–son relationship.

The interview information was extracted, coded, classified, and summarized into 17 themes, including 2 on the perception and attitude of the family members of patients in the terminal stage towards death, 5 themes about physical and psychological status of the respondents, 6 themes on needs of the respondents, and 4 themes about respondents’ responses to death and growth (Table 2).

**Table 2:** Qualitative analysis

Perception and attitude of the family members of patients in the terminal stage towards death	Physical and psychological status of the respondents	The needs of the respondents	Respondents’ responses to death and growth
Death is natural law	Uncomfortable	Painless	Feel At a loss
Death is scary	Fatigue	Euthanasia	Divert attention and seek a way out
	Lonely and helpless	Companionship and care for patients	Face life positively
	Anxious	Economic support	New understanding
	Lost and confused	Support from family	
		Effective communication with patients	

### Perception of and attitude towards death

Among the 10 family members of the patients in the terminal stage, 4 had the open-minded view on life and death, considering that birth, aging, illness, and death are tangible laws, and passing is a kind of relief for patients who are suffering. However, in terms of attitude, only 4 family members possessed a positive attitude, while the attitudes of the other 4 were negative; they considered that life and death are decreed by fate and cannot be prevented, only passively accepted. Therefore, of the family members of those who were about to pass away, the 4 who held a positive attitude expressed they could accept the death calmly, while the other 4 felt it would be painful and difficult to accept. Additionally, 2 other family members revealed their fear of death-related events and experiences.

#### Birth, aging, Illness, and death are natural laws

**Respondent A**: “Life and death are normal. The surgeon who performed the operation spoke directly to patients and asked us not to worry, saying everyone would die in the end. Although my child could not accept it, and I accepted it.” **Respondent B**: “I’m relatively optimistic. The fact is that more than 10,000 people suffer from this disease per day, and I have to be optimistic. But the doctor said the lifetime was too short, and it’s a little difficult for me to accept. I would find it even harder to accept if the patient passes away suddenly.” **Respondent E**: “We have to respect natural laws. Birth, aging, illness, and death are also natural laws. We should live healthily and face death calmly…” **Respondent F**: “I think that as long as you have done what you want to do, there is no regret in your whole life. I fell calm in the face of death.”

#### Death is terrifying

**Respondent C**: “Everyone will die one day, right? Now that he has suffered from this disease, he has to accept that’s his fate. I … never thought that he would have to encounter death so early, and I indeed cannot bear it. People say they are not afraid of death, but it is certain that they are actually afraid of death. In fact, all are afraid of death. We … never faced this issue when his mother passed away or his father had just passed away. But now, we have to face it and don’t want to see that, either. I still feel chilled when I think about it now. I said I would never want to see such a circumstance.” **Respondent D**: “I never thought about it before. I … never thought it would be so close to me. Life is especially vulnerable. The more you fear something, the more possibility it will happen. I think she is suffering, and it would be a relief for her to leave early, but I’m afraid of it.”

### Physical and psychological status of family members of patients in the terminal stage

Generally, patients with terminal cancer are in poor health. Cachexia and other conditions deprive patients of the ability to look after themselves. The conditions are recurrent and variable. The overwhelming majority of family members not only work hard to look after patients but also are under high stress, thereby threatening their physical and psychological health. In addition, this physical and psychological status is also influenced by many factors. For example, most family members supported patients throughout the entire progression of the disease until it was terminal. Faced with the fact that the patient is about to pass away, family members will feel powerless and frustrated. For some, their goal is to ensure the joy and happiness of their loved ones before death. Faced with the impending departure of the patient, the bereaved will have feelings of loneliness, helplessness, and confusion about their future lives.

In this study, 6 respondents experienced different degrees of physical fatigue and discomfort, and 6 respondents had different degrees and types of psychological experiences.

#### Physical discomfort

**Respondent A**: “I’m basically normal. I still have some edema. You look at my legs! I have found it the past week. I eat less here and only eat half a bowl of rice in the lunch box.” **Respondent C**: “I am now waiting for his death every moment. This process is painful. It’s hard, physically.” **Respondent D**: “In addition to anxiety and fatigue, I’m not adaptive in all other aspects. I once did not take food for 3 days at most.”

#### Fatigue

**Respondent A**: “My only idea was that I would go home and spend 2 months alone at home, and I wouldn’t see anyone after his death.” **Respondent D**: “What I experienced in the last 2 to 3 months is what I’ve experienced in the past half lifetime. I feel very tired now.” **Respondent H**: “I felt it was difficult to walk every day.”

#### Loneliness and helplessness

**Respondent A**: “I mainly felt lonely and was not depressed. I was worried, sad, and stressed because I was afraid of his pains.” **Respondent C**: “I asked an aunt previously, and she said that when she found out her husband was sick, she felt like the sky was falling. It is true. I also had such a feeling.” **Respondent G**: “Because I had no choice and had to face such matters, I was smoking during the period.” **Respondent H**: “I was afraid of being alone, and I did not dare to recall the past.”

#### Anxiety

**Respondent I**: “I am anxious. During the 11 years of my mother’s illness, including the 10 years of my father’s illness, I was anxious in every examination. Every time I got my mother’s CT, I was anxious and stressed.” **Respondent D**: “Since the illness, I was anxious in chemotherapy every time and even became more stressed during the re-examination.”

#### Loss and confusion

##### Respondent G

“I even did not want to wash my hair, and I had no desire to wash my hair and face. Now I … lose interest in many things, and I don’t know what to do. I have no driving force and suddenly lost … power.”

### Demands of family members of patients in the terminal stage

Every family member of a patient in the terminal stage has a strong desire to alleviate the patient’s physical pain, and the greatest hope is that the loved one can have a pain-free death. Most family members hope to accompany patients in the final stage of life and try their best to satisfy their requirements. Two family members mentioned euthanasia, hoping that it would be legislated in China as soon as possible. Families bearing huge economic pressures due to illness hope to obtain national economic security and medical resources. The support of family members and relatives can greatly relieve the physical and mental stresses of the caregivers of patients in the terminal stage.

#### No pain

**Respondent A**: “What was I worried about? Except for his pain, I was worried about nothing else. So, I told Dr. Guo I had only one request, that is, he did not feel pain.” **Respondent C**: “I did not want him to suffer pain, and I hoped that he passed out, unconscious.” **Respondent D**: “I felt he was too much in pain and suffering. If he could leave early, it would be a relief to him.” **Respondent E**: “I just pray that she will be able to get through this stage in peace. It would be better for her to leave sooner than let her suffer like this every day. I feel very uncomfortable when seeing her twitching and her expression every day.” **Respondent F**: “I want to tell the doctor not to let her suffer too much and to help her when medically permitted to ensure she has no pain for the rest of her life.”

#### Euthanasia

**Respondent E**: “I asked the doctor whether there was euthanasia in our country before I came here. When a patient is incurable, euthanasia is a relief for them.” **Respondent F**: “Even if there is a way to relieve pain, the law does not allow it. There is no euthanasia in China, right?”

#### Company and care of patients

**Respondent B**: “I want to accompany her more in the last stage of her life to avoid regret in the future.” **Respondent F**: “In my view, for cancer patients, the company of family members is especially important, because he certainly hoped his loved ones could accompany him. This is the most important thing.”

#### Economic support

**Respondent A**: “We are not very rich financially, but I still can bear the medical cost in this period according to what … Director Liang said. If the cost is higher, maybe I could not bear it.” **Respondent D**: “I thought it was too expensive, and I said at least I had to earn some living expenses.” **Respondent F**: “The economic burden is very heavy.”

#### Support from family and friends

**Respondent A**: “I still hope that she (daughter-in-law) could come back with the child because this benefits him (my son). After all, he sometimes calls the child’s name.” **Respondent B**: “I felt stressed at first. After sharing with my brothers and sisters, I was a little less stressed.” **Respondent C**: “After my elder brother and my younger brother came, I felt a little more relaxed. At least they could arrange his funeral. It is a good thing to have brothers and sisters.”

#### Effective communication with patients

**Respondent A**: “We do not communicate with each other. Our communication basically ended in failure. I don’t know what he asks me right. I would convey what the doctor said to him. This is my attitude towards him.” **Respondent B**: “When I was watching videos, she always thought I was watching TV plays and would not be happy, maybe due to pain. So, I tried my best to watch videos when she was asleep.” **Respondent D**: “I think we lack communication and always quarrel. I always wanted to engage in the industry, but I just couldn’t keep up with it in terms of energy, so I quit[ted]. My wife still thinks I’m working in the industry.” **Respondent I**: “I don’t have any regrets about my mother. My mother is also calm. She knew she was suffering from the disease. We didn’t hide anything from her.”

### Response and growth of family members of patients in the terminal stage

Faced with the trauma caused by the impending death of their family members, the respondents recognized the importance of diverting attention and releasing negative emotions. One respondent even considered seeking help from professionals. Furthermore, the respondents realized that although they might have lost some roles (such as wife and father), they still had other important and valuable existing roles. Two respondents expressed that their outlook on life had changed considerably. Almost all respondents said that as time went on, they could recover from the sorrow of losing their loved ones, return to normal life, and seek new meaning in their lives.

#### Bewilderment

**Respondent A**: “I don’t know what I should do.” **Respondent D**: “I don’t know how to face our kids, and I also feel really bad. But there is no way. I really do not know how to vent my emotions and do not know to whom I should pour out.” **Respondent G**: “The child just entered … primary school, and the grandma insisted that she saw off her mother. I do not know whether it is right or not.”

#### Distracting attention and seeking a release

**Respondent A**: “I plan to live with my daughter for a period or look after her baby. That’s all.” **Respondent C**: “I’m going to go for a walk and try not to stay at home. Sometimes, we need to vent our sadness. We may go to a lonely place to cry or shout.” **Respondent D**: “I will adjust myself, and I want to go to the baptism.”

#### Being positive about life

**Respondent B**: “I have a strong adaptive ability. It’s inevitable. Take your time.” **Respondent C**: “We have to be strong. You may be suffering in the future in case of illness.” **Respondent E**: “I can treat it correctly! The dead have rested in peace, and the living should live well.” **Respondent F**: “It must be painful to lose kinsfolks, but the living should live well. You must face it and adjust yourself. Others cannot help you.”

#### New understandings

**Respondent B**: “I seem to be flexible. Sometimes, I feel I should be kind to myself. In fact, if you get ill, you drag down others, which is to the disadvantage of your health. More importantly, you have to endure it.” **Respondent D**: “He passed away. For me, some things ended, and some things just started.”

## Discussion

3.1 Through qualitative interviews, it was found that most family members had heavy physical and psychological burden during the period of caring for terminally ill patients. Lack of effective communication and emotional exchange between some family members and patients; Some family members can get the support of family members and relatives to a certain extent, but individual family members lack the corresponding support, and lack the corresponding channels of counseling. In this case, the family members choose to look down at the phone and take comfort from the phone. The after-effect mechanism of this bowing behavior of family members is the codependency theory, which focuses on the mutual influence between people’s behaviors. That is, the emotions, perceptions, or behaviors of the dying patient can affect the emotions, perceptions, or behaviors of the family members, and this influence in turn continues to affect both parties. This behavior is more common in practical work, should cause the attention of nursing staff and should give timely intervention. Of course, grief can bring us not only negative reactions, but also positive reactions, which can make people grow up and re-understand death [11]. This can also be seen through the results of five assessments conducted by the “psychological pain thermometer”, while the care experience in the terminal stage brings negative effects on family members such as stress, burden, fatigue and depression. It also enables the caregiver to grow and gain new insights.

3.2 Studies have found that the experience of caring for patients with nurses leaves a deep impression on family members, who want to be able to talk about their feelings to a familiar nurse [12]. Therefore, in the terminal stage of patients, nurses should observe and understand the communication between patients and their families on the basis of daily nursing work, give timely guidance and assistance to their families, and encourage the courage to express their inner emotions. It is suggested that clinical nurses should implement the care of the family members of patients with malignant tumors from an integrated perspective, such as creating a warm and humanistic care environment, creating a special ecological environment in the ward, and fully considering the social needs of the family members.

After the death of patients, nurses should remain in touch with the bereaved family members, conduct household follow-ups to understand the current situation of the bereaved in time, consider the emotional experience of the bereaved after the patient’s death, encourage them to express their sorrow, actively listen to them, and help them seek an appropriate coping method. Additionally, Chinese researchers [13] have confirmed that providing the caregivers of patients with terminal-stage cancer active health education, psychological nursing, family support, nursing and professional assistance, and grief counseling as well as improving their coping skills can alleviate the psychological pain and sadness of deceased patients’ family members and improve their quality of life.

3.3 Grief counseling has been well established in other countries; a complete three-level grief-support conceptual model of palliative care [14], a grief risk screening tool [15], and a grief counseling team [16] have been developed. Foreign countries provide not only palliative care for patients but also bereavement support for the family members of those patients, which can promote the adaptation of family members to bereavement and reduce the risk of prolonged grief disorder [17]. However, there is still no complete system, clear scheme, or reasonable and normative guidance for grief counseling in China. Domestic and international experts do not determine the timing of the start of counseling, and different countries have diverse opinions on the definition of the terminal stage. Although the timing of the start differs, the common point is that all focus on the continuity of the counseling process before and after death [18]. In this study, although the psychological pain thermometer was used to measure at different time nodes, and the measured value decreased in a step by step manner, it is highly likely that the changes in the grief experience of family members will gradually fade with the passage of time because no professional psychological counseling measures have been carried out. Therefore, the significance of complete and continuous counseling for family members after the death of patients in the future remains to be studied.

## Conclusion

Through the exploration of the grief experience of the families of patients with malignant tumors, it is suggested that nursing staff should provide physical, psychological, social and spiritual care for the families of patients with cancer, help the families of patients with cancer re-establish life goals, return to society as soon as possible, and reduce the burden of society and family, which are worthy of our attention and research content. We look forward to proposing grief counseling methods suitable for our national conditions and establishing our grief prevention, management and treatment system in the future.

## Data Availability

All data generated or analysed during this study are included in this article. Further enquiries can be directed to the corresponding author.
